# Synthesis and physical properties characterization of artificial seawater samples

**DOI:** 10.1016/j.dib.2026.112759

**Published:** 2026-04-07

**Authors:** Ana Rousseva, Ali M. Alasmari, Ratul Das

**Affiliations:** a 4700 King Abdullah University of Science and Technology, Thuwal 23955, Saudi Arabia; b Acwa, Building 24, Innovation Cluster 3-3465, 4700 King Abdullah University of Science and Technology, Thuwal 23955, Saudi Arabia

**Keywords:** Artificial seawater, Salinity, Desalination, Density, Ion composition

## Abstract

This dataset comprises synthesized artificial seawater samples prepared in accordance with ASTM D1141–98 and spanning an extended salinity range from 36,052 to 80,027 ppm. The samples reproduce the major ionic composition of seawater (Na⁺, Ca²⁺, Mg²⁺, K⁺, Sr²⁺, Cl⁻, Br⁻, F⁻, HCO₃⁻, CO₃²⁻, SO₄²⁻, and B(OH)₄⁻), with constant inter‑ionic molar ratios maintained above standard seawater salinity through a total dissolved solids (TDS) scaling approach. Carbonate and borate species were handled separately because their aqueous speciation is pH‑dependent. For each synthesized solution, experimentally measured physical properties are reported, including density (36,052–46,000 ppm), electrical conductivity, pH, and osmolality, from which osmotic pressure may be derived. Measurements are provided together with detailed preparation metadata, including stock solution volumes, scaling factors, and dilution procedures, allowing absolute salinity (SA) to be explicitly defined for every sample and directly linked to the measured properties. To validate the synthesized ionic composition, inductively coupled plasma optical emission spectroscopy (ICP‑OES) measurements of Na, Mg, Ca, K, Sr, S, and B were performed at selected salinities spanning the full range (36,052–80,027 ppm). Comprehensive documentation of sample preparation, analytical protocols, and measurement conditions is included to ensure reproducibility. The dataset enables derivation of empirical relationships between absolute salinity (or TDS) and key thermophysical properties, supports evaluation of seawater property models extrapolated to elevated salinities, and provides high‑quality reference data for laboratory calibration. Potential applications include desalination process modeling, sensor development, instrument validation, and assessment or extension of thermodynamic and empirical seawater property frameworks under high‑salinity conditions relevant to brines and engineered seawater systems.

Specifications Table

**Table:** 

Subject	Earth & Environmental Sciences
Specific subject area	*Sea and saline water physical properties*
Type of data	Tables, Graphs, and Figure Raw and Processed
Data collection	Artificial seawater solutions with total dissolved solids (TDS) ranging from 36,052 to 80,027 ppm were synthesized in accordance with ASTM D1141‑98 using gravimetric salt preparation and volumetric dilution with deionized water. Density was measured using an Anton Paar DMA 5000 M densitometer, osmolality using a VAPRO® 5600 vapor‑pressure osmometer, and pH and electrical conductivity using Fisherbrand Accumet AB150 and Hach HQ40d instruments equipped with PHC101 (pH) and CDC401 (EC) probes at 25 °C. Ionic composition (Na, Mg, Ca, K, Sr, S, and B) was validated by ICP‑OES following 0.45 µm filtration and dilution in 1% (v/v) HNO₃.
Data source location	Acwa*, Building 24,* King Abdullah University of Science and Technology*, Thuwal, Makkah, Saudi Arabia*
Data accessibility	Repository name: Synthesis and Physical Properties Characterization of Artificial Seawater Samples Ranging from 36,052 ppm (Standard Seawater) to 80,027 ppm Data identification number: 10.17632/jx9kxvcymm.1
Related research article	*None*

## 1 Value of the Data

• The dataset provides experimentally measured physical and chemical properties of artificial seawater across an extended salinity range (36,052–80,027 ppm), exceeding typical oceanic conditions and encompassing concentrations relevant to desalination brines and laboratory calibration standards.
• Absolute salinity is explicitly defined for all samples through controlled synthesis and documented scaling factors, enabling direct and traceable relationships between salinity and measured properties, including density, electrical conductivity, pH, osmolality, and ionic composition.
• The inclusion of ICP‑OES–measured concentrations of major and minor ions (Na, Mg, Ca, K, Sr, S, and B) allows independent verification of solution composition and supports reuse of the dataset for analytical calibration, method validation, and quality control.
• The data can be reused to develop, validate, or refine empirical correlations (e.g., EC–TDS, salinity–density), evaluate thermodynamic seawater property models at elevated salinities, or benchmark laboratory instruments operating outside standard seawater ranges.
• The dataset is directly applicable to desalination research, including process modeling, sensor development, and physical property estimation for feed, brine, and permeate streams, as well as to broader studies in marine chemistry and solution thermodynamics [3].
• By enabling robust correlations between readily measured bench‑top parameters (electrical conductivity, pH, density, and osmolality) and absolute salinity—with validation from selected density and ICP‑OES measurements—the dataset provides actionable value for desalination operators, oceanographic researchers, and coastal monitoring programs relying on rapid, instrument‑based assessments of seawater composition [3].

## 2 Background

Accurate laboratory data describing seawater properties across a wide salinity range are required for instrument calibration, model development, and engineering calculations. Standardized thermodynamic frameworks such as TEOS-10 rely on experimental data combined with theoretical extensions to represent seawater behavior, particularly at salinities higher than those typically encountered in the open ocean [1]. However, experimental datasets validating seawater physical and chemical properties at elevated salinities remain limited.

Artificial seawater prepared according to ASTM D1141–98 provides a reproducible basis for generating reference solutions with known ionic composition [2]. By applying controlled total dissolved solids (TDS) scaling while maintaining constant inter-ion ratios, artificial seawater can be extended beyond standard salinity while preserving chemical consistency. Independent chemical verification, such as ICP-OES analysis, is necessary to confirm that synthesized solutions match intended compositions.

This dataset was compiled to document the preparation, physical characterization, and chemical validation of artificial seawater samples spanning standard to high salinity conditions. The data article provides full access to measured properties and preparation details, enabling reuse independent of any specific modeling or interpretive framework.

## 3 Data Description

A data repository containing detailed sample preparation protocols, physical property measurements, and elemental composition analyses for artificial seawater solutions.

### 3.1 Repository structure

**01_Sample_Preparation/**

Documentation and detailed preparation recipes for synthesizing artificial seawater samples across multiple total dissolved solids (TDS) concentrations.

•**exact_salt_composition.csv** - Exact salt composition and concentrations used to prepare artificial seawater samples across the investigated salinity range. The table provides a detailed breakdown of individual salt components and their exact concentrations for each sample, covering total dissolved solids (TDS) levels from 36,052 ppm (standard seawater) to 80,027 ppm.

**02_Physical_Properties/**

Physical and chemical characterization data for samples across different TDS ranges.

• **density_averages_by_tds_vs_temperatures.csv** - Average density of artificial seawater samples as a function of TDS concentration and temperature (36,052–46,013 ppm).
• **density_trials_data.csv** - Replicate density measurements for artificial seawater samples at different TDS concentrations (36,052–46,013 ppm), used to assess repeatability.
• **DensityVsTemperatureAtTDS.svg** - Density variation with temperature for artificial seawater samples at different TDS levels.
• **ec_and_pH_by_tds.csv** - Electrical conductivity (EC) and pH of artificial seawater samples as a function of TDS (36,052–80,027 ppm).
• **osmolality_data.csv** - Osmolality measurements of artificial seawater samples across a wide TDS range (36,052–80,027 ppm).
• **OsmolalityVsTDS.svg** - Relationship between osmolality and total dissolved solids (TDS) for prepared samples.
• **pHvsTDS.svg** - Variation of pH with TDS for artificial seawater samples.
• **physical_properties_summary_overall.csv** - Summary of measured physical and chemical properties for all artificial seawater samples.

**03_ICP-OES/**

Inductively Coupled Plasma Optical Emission Spectroscopy (ICP‑OES) elemental analysis for selected artificial seawater samples across the investigated salinity range (36,052–80,027 ppm).

• **B Expected vs. ICP-OES.svg** - Comparison of expected and measured boron concentrations obtained by ICP‑OES across selected TDS levels.
• **Ca Expected vs. ICP-OES.svg** - Comparison of expected and measured calcium concentrations determined by ICP‑OES.
• **ICP-OES_by_tds.csv** - Elemental composition of artificial seawater samples measured by ICP‑OES, organized by TDS concentration.
• **ICP-OES_spikes.csv** - ICP‑OES spike recovery results used for analytical method validation and quality control.
• **K Expected vs. ICP-OES.svg** - Comparison of expected and measured potassium concentrations from ICP‑OES analysis.
• **Mg Expected vs. ICP-OES.svg** - Comparison of expected and measured magnesium concentrations across the investigated TDS range.
• **Na Expected vs. ICP-OES.svg** - Comparison of expected and measured sodium concentrations determined by ICP‑OES.
• **S Expected vs. ICP-OES.svg** - Comparison of expected and measured sulfur concentrations obtained by ICP‑OES.
• **Sr Expected vs. ICP-OES.svg** - Comparison of expected and measured strontium concentrations from ICP‑OES analysis.

## 4 Experimental Design, Materials and Methods

### 4.1 Overall experimental design

Artificial seawater samples were prepared over a salinity range of 36,052–80,027 ppm according to ASTM D1141‑98. Two concentrated stock solutions containing the major seawater salts were first prepared and subsequently diluted to obtain all target salinities. Salinities above standard seawater were achieved by applying a total dissolved solids (TDS) scaling factor while maintaining constant molar ratios among dissolved ions, except for carbonate and borate species, whose speciation is pH‑dependent.

For each target salinity, three independent replicate solutions were prepared. All replicates were characterized in terms of electrical conductivity, pH, and osmolality. Selected salinities (36,052; 40,045; 45,004; 50,012; 60,029; 70,022; and 80,027 ppm) were further analyzed by ICP‑OES to validate ionic composition. Density measurements were conducted separately on a subset of samples within the 36,052–46,013 ppm range. All measurements were performed under controlled temperature conditions, and deionized water from a single source was used throughout to ensure consistency.

### 4.2 Stock solution preparation

Two artificial seawater stock solutions were prepared in accordance with ASTM D1141‑98. Each stock solution was synthesized to a final volume of 1 L to minimize relative weighing and volumetric errors.

Individual salts were weighed using a high‑precision analytical balance (±0.001 g). Each salt was placed in a separate plastic weighing boat, which was subsequently rinsed with deionized water to ensure quantitative transfer of all solids. This step was particularly important for hydrated salts (e.g., magnesium chloride hexahydrate). The weighed salts and rinse water were transferred into one of two 250 mL round‑bottom flasks corresponding to the respective stock solution.

After all salts were added, approximately 100 mL of deionized water was introduced into each flask. Sterilized magnetic stir bars were added, and the flasks were sealed with sterilized glass stoppers. The flasks were placed on magnetic stir plates and stirred until all visible salt crystals had dissolved, indicating complete dissolution.

Each stock solution was then transferred into a 1 L measuring cylinder that had been cleaned with laboratory detergent, rinsed with ethanol, and thoroughly rinsed with deionized water. The round‑bottom flasks were rinsed with an additional 100 mL of deionized water, which was combined with the solution in the measuring cylinder. The solutions were topped up to the 1 L mark with deionized water, transferred to 1 L glass media bottles, and stirred vigorously using large sterilized magnetic stir bars to ensure complete homogeneity.

### 4.3 Artificial seawater sample preparation

Final artificial seawater samples were prepared at target TDS values of 36,052; 38,206; 39,095; 40,045; 41,004; 42,013; 43,022; 44,031; 45,004; 46,013; 47,022; 48,031; 49,004; 50,012; 55,021; 60,029; 65,002; 70,022; 75,019; and 80,027 ppm. Three independent replicate solutions were prepared for each salinity.

Prior to sample preparation, 100 mL glass media bottles were cleaned with laboratory detergent, rinsed with ethanol, and thoroughly rinsed with deionized water. For each sample, NaCl and anhydrous Na₂SO₄ were weighed separately using the same analytical balance employed for stock solution preparation, based on the applied TDS scaling factor relative to standard seawater. Each weighing boat was rinsed with deionized water to ensure quantitative transfer, and the salts and rinse water were transferred into the corresponding glass bottle, followed by the addition of 20 mL of deionized water.

A sterilized magnetic stir bar was added, and the solution was stirred until all salt crystals had completely dissolved. While stirring, measured volumes of the two stock solutions were added sequentially using freshly calibrated micropipettes (1–10 mL, 100–1000 µL, or 1–100 µL, depending on the required volume). Stock solution 1 was added first, followed by stock solution 2, in accordance with ASTM D1141‑98 to avoid salt precipitation.

After stock addition, the solution was stirred for approximately 5 min to ensure mixing. The contents were then transferred to a 100 mL measuring cylinder, the glass bottle was rinsed with deionized water, and the rinsate was added to the cylinder. The solution was diluted to the 100 mL mark with deionized water, returned to the glass bottle, and briefly vortexed to ensure complete homogeneity.

### 4.4 Temperature control

Prior to physical property measurements, all samples were equilibrated to 25 °C using a temperature‑controlled water bath. Sample bottles were submerged to a level below the cap to prevent ingress of bath water. Samples remained in the bath until thermal equilibrium was achieved, as confirmed by stabilization of the measured solution temperature at 25 °C.

### 4.5 Electrical conductivity and pH measurements

pH and electrical conductivity were measured using two pH meters (Fisherbrand Accumet AB150 and Hach HQ40d equipped with a PHC101 probe) and one conductivity probe (Hach HQ40d with CDC401 probe). pH probes were calibrated daily using standard buffer solutions at pH 7.01 and 10.01.

Electrical conductivity measurements were performed twice for each sample: first using the existing instrument calibration and subsequently after a custom calibration with 50,000 and 80,000 mg L⁻¹ conductivity standards. Prior to each measurement, probes were rinsed with deionized water, gently dried, and immersed perpendicular to the base of the sample bottle. Readings were recorded after signal stabilization, defined as three consecutive identical measurements at 25 °C.

### 4.6 Osmolality measurements

Osmolality was measured using an EliTechGroup VAPRO® Vapor Pressure Osmometer (Model 5600). For each replicate, three independent measurement trials were performed. Instrument performance checks and calibration were conducted daily in accordance with the manufacturer’s instructions. Samples were loaded and analyzed following the standard operating procedures specified by the manufacturer.

### 4.7 Density measurements

Density measurements were performed using an Anton Paar DMA 5000M vibrating‑tube densitometer over a controlled temperature range. For each replicate solution, three independent density measurements were conducted. Daily air and deionized water checks were carried out prior to sample analysis to verify instrument performance. Density data were collected for samples with TDS values of 36,052; 38,206; 39,095; 40,045; 41,004; 42,013; 43,022; 44,031; 45,004; and 46,013 ppm.

### 4.8 ICP-OES sample preparation

Selected artificial seawater samples (36,052; 40,045; 45,004; 50,012; 60,029; 70,022; and 80,027 ppm) were analyzed by inductively coupled plasma optical emission spectroscopy (ICP‑OES) to validate ionic composition. Samples with salinity below 55,000 ppm were filtered through 0.45 µm syringe filters prior to dilution. All samples were diluted sequentially to a final dilution factor of 500× (10×, 10×, 5×) using deionized water containing 1% (v/v) HNO₃.

Boron and strontium were additionally analyzed at lower dilution factors (5× and 10×) to assess analytical performance and sensitivity. All diluted samples were prepared using calibrated micropipettes to ensure volumetric accuracy.

### 4.9 ICP-OES calibration, loading, and analysis

Calibration standards were freshly prepared prior to each ICP‑OES analytical run. Calibration concentrations of 1, 10, and 100 ppm were used for Na, Mg, Ca, K, and S, while concentrations of 0.1, 1, and 10 ppm were used for Sr and B. Samples and standards were arranged in the autosampler tray in order of increasing TDS to minimize potential carryover effects. Blank and quality‑control samples were analyzed at the beginning of each run and after every ten samples; boron blanks were included at higher frequency to account for its lower concentration range.

Emission lines were selected based on peak shape, signal stability, relative standard deviation, and calibration linearity. Measurements were performed in both axial and radial viewing modes where applicable to ensure optimal sensitivity and accuracy.

### 4.10 ICP-OES spiking procedure

Artificial seawater samples were independently spiked with known concentrations of individual cations using prepared spike solutions. Spike solutions were prepared gravimetrically, diluted as required, and added to artificial seawater at a fixed volumetric ratio prior to final dilution. Spiked samples and corresponding spike solutions were analyzed under the same ICP‑OES operating conditions as the unspiked samples to evaluate analytical accuracy and recovery.

## Limitations

Due to time constraints and the practical difficulty of reproducing the full sample set, density measurements were limited to samples with TDS values up to 46,000 ppm.

## Ethics Statement

This study involved only laboratory synthesis and physicochemical measurements of artificial seawater and did not involve human participants, personal data, or animals; therefore, ethics committee approval and informed consent were not required. No field sampling or collection of protected materials was performed. All experimental work complied with institutional EHS policies, including appropriate PPE, chemical handling, and waste disposal. The research does not present dual‑use or biosecurity concerns.

## CRediT Author Statement

**Ana Rousseva:** Conceptualization, Data curation, Formal Analysis, Investigation, Methodology, Validation, Visualization, Writing – original draft; **Ali Mohammed Alasmari:** Validation, Visualization, Writing – original draft; **Ratul Das:** Conceptualization, Project administration, Supervision, Writing – review & editing.

## Data Availability

Mendeley DataSynthesis and Physical Properties Characterization of Artificial Seawater Samples Ranging from 36,052 ppm (Standard Seawater) to 80,027 ppm (Original data).
